# Supplementary material for: The evolution of metastatic upper tract urothelial carcinoma through genomic-transcriptomic and single-cell protein markers analysis
Source: Nat Commun. 2024 Mar 18;15:2009. doi: 10.1038/s41467-024-46320-w (PMC10948878; doi:10.1038/s41467-024-46320-w)
Supplement: Supplementary file 1 — Supplementary Information [file 41467_2024_46320_MOESM1_ESM.pdf]

Supplementary Figure 1. Comparison of microsatellite instability (MSI) and total mutational burden (TMB) between primary and metastatic upper tract urothelial carcinoma (UTUC) (n = 24 and 18).

Supplementary Figure 2. Timeline of 7 patients with paired primary and metastatic UTUC. Triangles represent tumor sampling. Each rectangle shows a treatment period of chemotherapy and/or immunotherapy.

Supplementary Figure 3. Comparison of genomic alterations between paired primary and metastatic UTUC.

Supplementary Figure 4. A heatmap of Gene Set Enrichment Analysis (GSEA) enrichment scores using RNA-seq data in primary and metastatic UTUC (n = 6 and 11).

Supplementary Figure 5. Results of CIERSORT analysis using RNA-seq data in primary and metastatic UTUC (n = 1 and 9).

Supplementary Figure 6. Comparison of cellular abundance between immune-depleted and immune-inflamed samples.

Supplementary Figure 7. Intrapatient heterogeneity of tumor phenotype and immune environment in paired primary and metastatic UTUC.

Supplementary Table 1. Patient and sample characteristics.

Supplementary Table 2. Immune-related genes used by RNA-seq based classifier

Supplementary Table 3. A list of the metal-labeled antibodies used in Imaging mass cytometry.

Supplementary Table 4. Exome sequencing statistic summaries.

Supplementary Figure 1

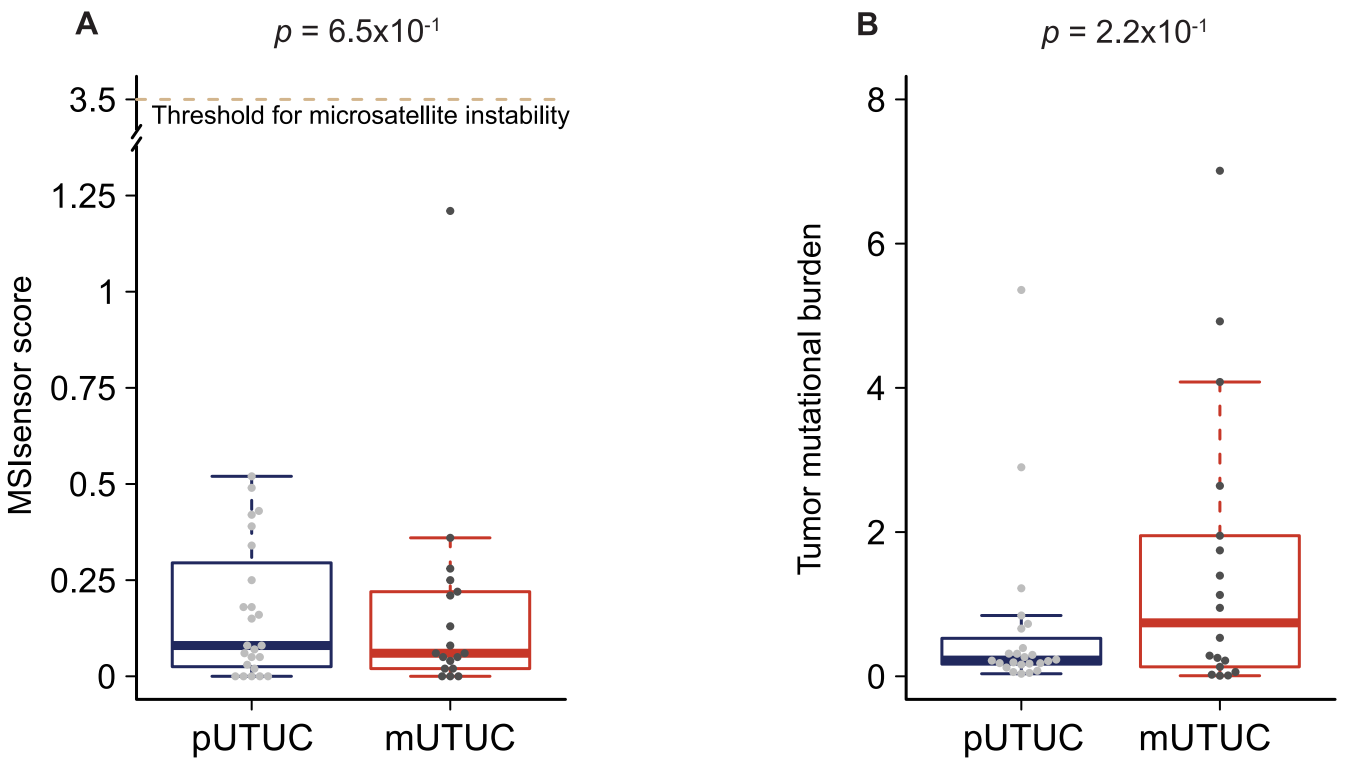

Supplementary Figure 1. Comparison of microsatellite instability (MSI) and total mutational burden (TMB) between primary and metastatic upper tract urothelial carcinoma (UTUC) (n = 24 and 18). (a) Box plots showing MSIsensor scores. The cut-off for defining MSI-high versus MS stable samples was 3.5 (MSI-High > 3.5, MS stable < 3.5). (b) Box plots showing TMB burden. Centers, boxes, and whiskers indicate medians, quantiles, and minima/maxima, respectively. Data points beyond the whiskers are potential outliers. Two-sided Mann–Whitney *U*-test without multiple testing correction was used for comparison. pUTUC, primary UTUC; mUTUC, metastatic UTUC. Source data are provided as a Source Data file.

Supplementary Figure 2

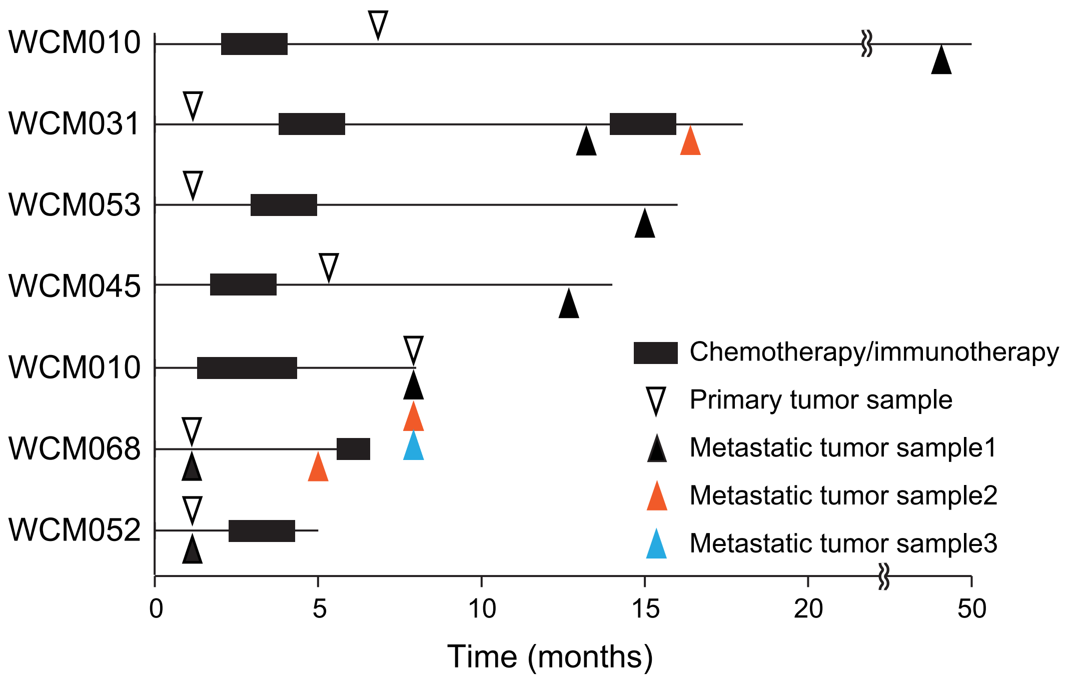

Supplementary Figure 2. Timeline of 7 patients with paired primary and metastatic UTUC. Triangles represent tumor sampling. Each rectangle shows a treatment period of chemotherapy and/or immunotherapy.

Supplementary Figure 3

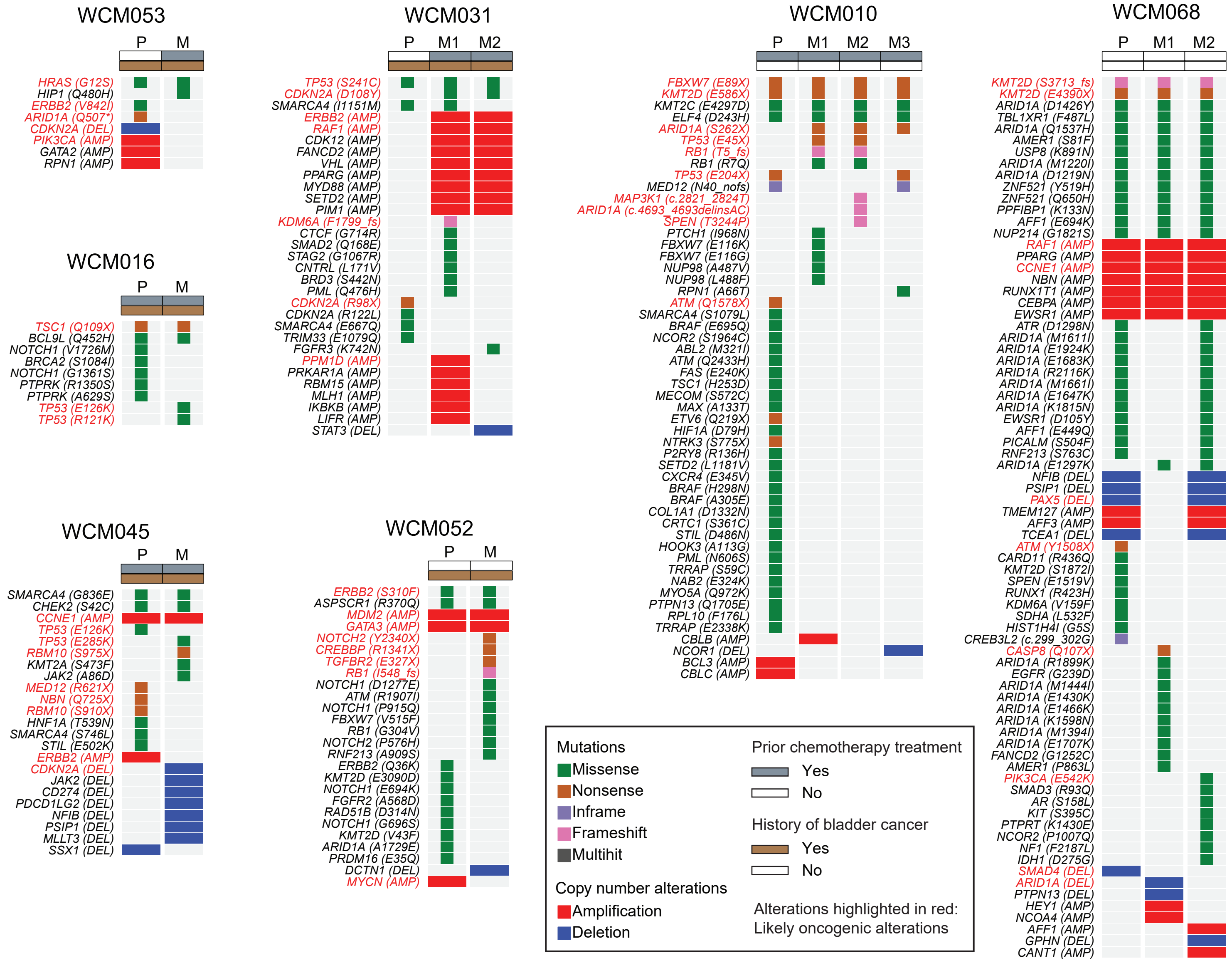

Supplementary Figure 3. Comparison of genomic alterations between paired primary and metastatic UTUC. Oncoprints showing somatic mutations (green), amplification (red), and deletion (blue) of cancer genes in primary (P) and matched metastatic (M) UTUC obtained from 7 patients. Discordance in likely oncogenic alterations between primary tumor and paired metastases is shown in red. Source data are provided as a Source Data file.

Supplementary Figure 4

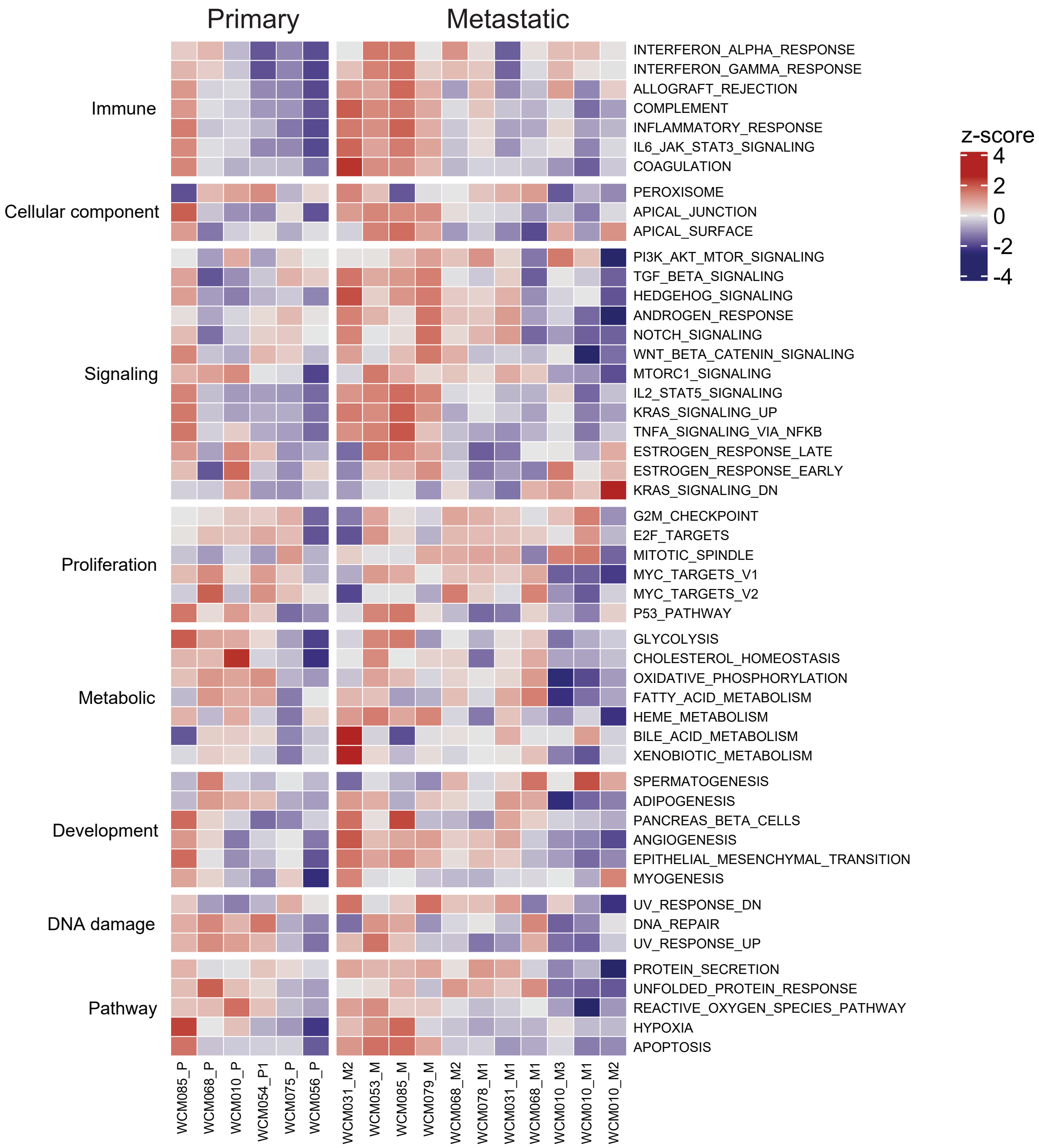

Supplementary Figure 4. A heatmap of Gene Set Enrichment Analysis (GSEA) enrichment scores using RNA-seq data in primary and metastatic UTUC (n = 6 and 11). No significant enrichment of biological gene sets is shown between primary and metastasis (Wilcoxon signed-rank test, FDR > 0.25). Source data are provided as a Source Data file.

Supplementary Figure 5

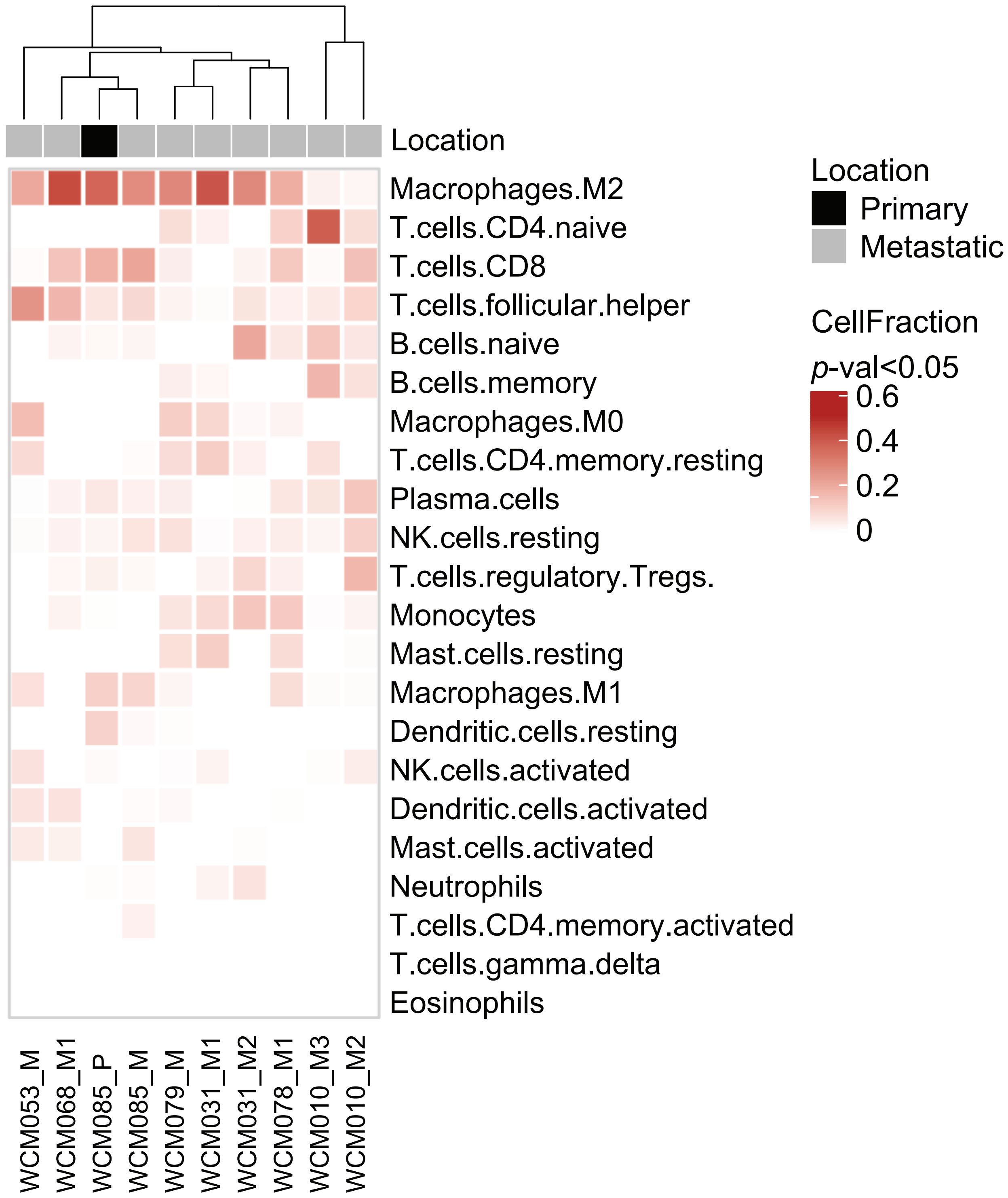

Supplementary Figure 5. Results of CIERSORT analysis using RNA-seq data in primary and metastatic UTUC (n = 1 and 9). Samples whose empirical *p*-value for the deconvolution was over 0.05 were removed. Source data are provided as a Source Data file.

Supplementary Figure 6

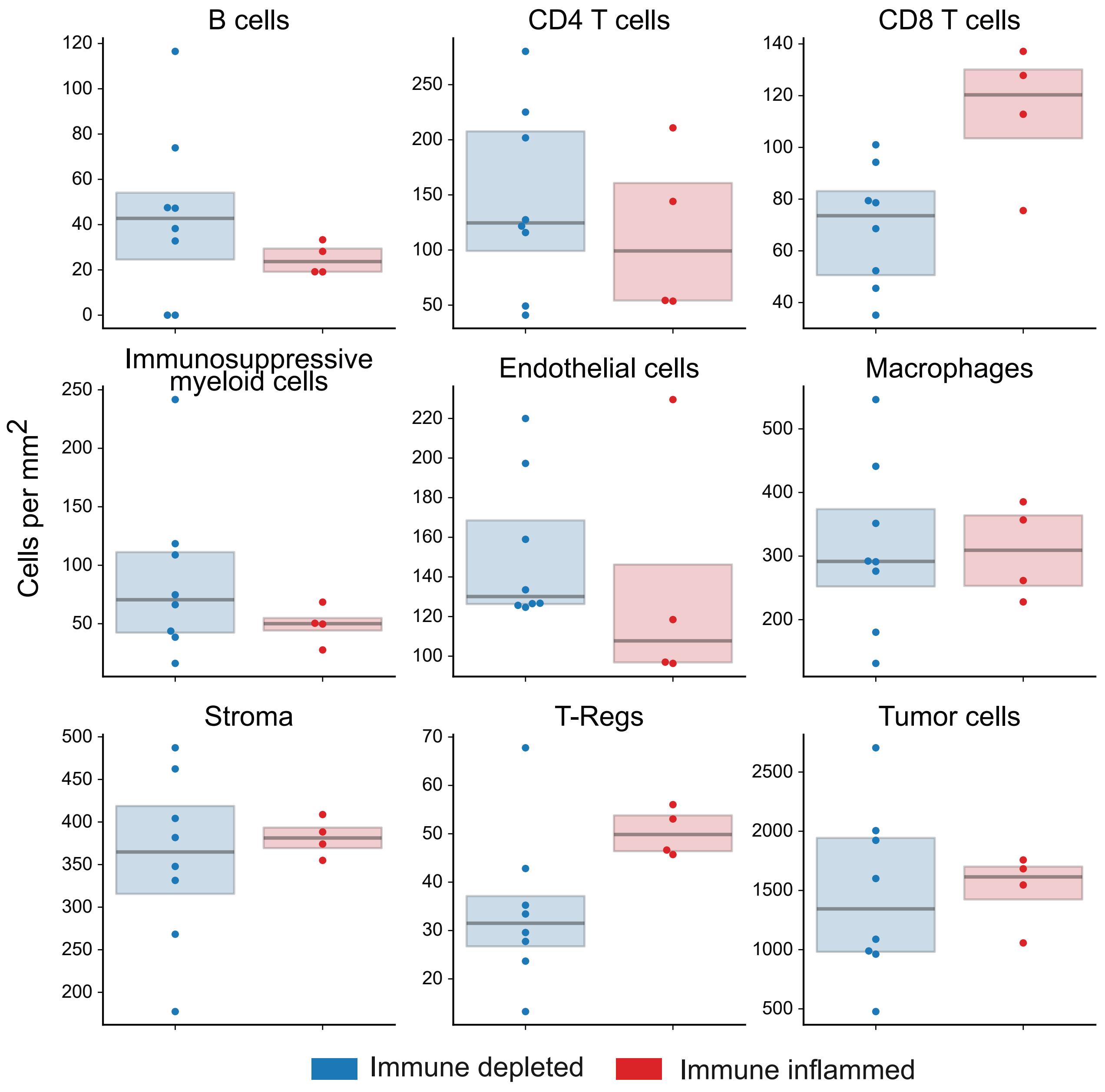

Supplementary Figure 6. Comparison of cellular abundance between immune-depleted and immune-inflamed samples.
Abundance of cells analyzed by Imaging Mass Cytometry between samples classi-fied as immune-inflamed (4 samples from 3 patients) by the RNA-seq-based classifi-er and those as immune-depleted (8 samples from 4 patients). Each point represents the normalized abundance of each cell type in each sample. Boxplot centers and box edges indicate medians and 25, and 75 quantiles, respectively. Source data are provided as a Source Data file.

Supplementary Figure 7

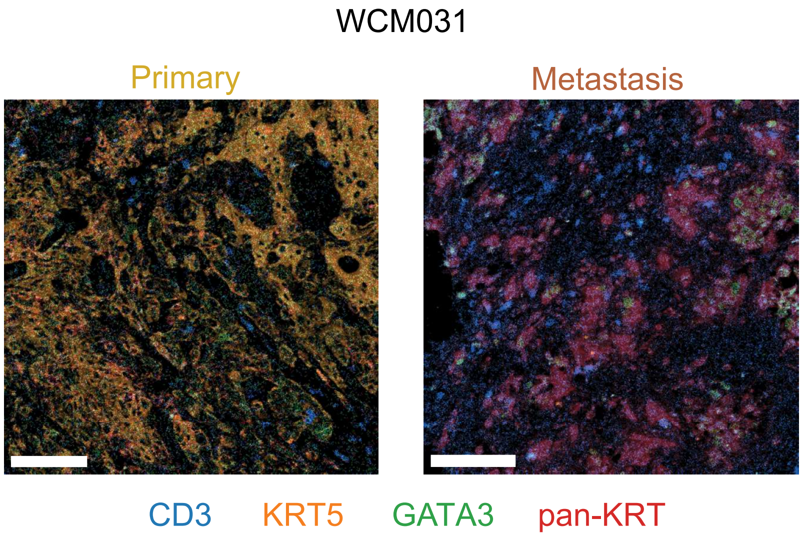

	Primary	Metastasis
Ba/Sq vs Lum (log ratio)	0.83	-1.25
CD8+ T-cells (per mm2)	78.6	134.5
Phenotype predicted	Ba/Sq, Depleted	Lum, Inflammed

Supplementary Figure 7. Intrapatient heterogeneity of tumor phenotype and immune environment in paired primary and metastatic UTUC. Regions of interest (ROI) for samples of patient WCM031 illustrating differential tumor phenotype and immune infiltration between primary and metastasis. Primary tumor cells show cytoplasmic staining of the basal/squamous (Ba/Sq) marker KRT5. Metastatic tumor cells demonstrate nuclear staining of the luminal (Lum) marker GATA3. CD3 staining highlights low T-cell infiltration (depleted) in the primary tumor and high T-cell infiltration (inflamed) in the metastatic tumor. The images are representative of 2 ROIs from primary and 3 ROIs from metastasis. Numeric data of primary and metastatic tumors is a mean value of 2 and 3 ROIs in single experiment. Scale bar, 100 μm.

Supplementary Table 1. Patient and sample characteristics.

Case.ID	Sample.ID	WES	RNA-seq	IMC	Tissue coll	Primary/M	Gender	Smoking h	Chemothe	Immunoth	Prior histo	pT	CLONET_P	Histology_	Consensus	T-cell sigat	Mutation_	TMB_SCORE
WCM006	WCM006_	Y	N	N	Renal pelv	Primary	Female	Former	N	N	N	pT4	55	70	NA	NA	344	2.898506
WCM010	WCM010_	Y	Y	Y	Lymph noc	Metastasis	Female	Former	Y	Y	N	NA	98	90	LumU	Depleted	167	1.396217
WCM010	WCM010_	Y	Y	Y	Lymph noc	Metastasis	Female	Former	Y	Y	N	NA	73	80	LumP	Depleted	151	1.128191
WCM010	WCM010_	Y	Y	Y	Lymph noc	Metastasis	Female	Former	Y	Y	N	NA	85	80	LumP	Depleted	130	0.949336
WCM010	WCM010_	Y	Y	Y	Renal pelv	Primary	Female	Former	Y	Y	N	pT1	95	80	LumP	Depleted	492	5.357383
WCM016	WCM016_	Y	N	N	Lung	Metastasis	Male	Former	Y	N	Y	NA	57	60	NA	NA	55	0.255722
WCM016	WCM016_	Y	N	N	Renal pelv	Primary	Male	Former	Y	N	Y	pT1	50	70	NA	NA	123	0.31435
WCM018	WCM018_	Y	N	N	Lung	Metastasis	Male	Former	N	N	Y	NA	47	80	NA	NA	397	0.131021
WCM019	WCM019_	Y	N	N	Ureter	Primary	Male	Former	N	N	Y	pT3	NA	70	NA	NA	398	0.058842
WCM022	WCM022_	Y	N	N	Renal pelv	Primary	Male	Former	Y	N	N	pT1	36	90	NA	NA	16	0.16232
WCM031	WCM031_	Y	Y	Y	Liver	Metastasis	Female	Former	Y	N	Y	NA	65	50	Stroma-ric	Depleted	164	4.081081
WCM031	WCM031_	Y	Y	N	Liver	Metastasis	Female	Former	Y	N	Y	NA	NA	30	LumU	Depleted	5	0.00791
WCM031	WCM031_	Y	N	Y	Renal pelv	Primary	Female	Former	N	N	Y	pT3	24	80	NA	NA	29	0.391892
WCM045	WCM045_	Y	N	N	Mediastinu	Metastasis	Male	Former	Y	N	Y	NA	NA	30	NA	NA	88	1.746146
WCM045	WCM045_	Y	N	N	Renal pelv	Primary	Male	Former	Y	N	Y	pT4	NA	30	NA	NA	102	0.727383
WCM052	WCM052_	Y	N	N	Lymph noc	Metastasis	Male	Former	N	N	Y	NA	70	70	NA	NA	86	0.286288
WCM052	WCM052_	Y	N	N	Ureter	Primary	Male	Former	N	N	Y	pT1	84	80	NA	NA	97	0.296847
WCM053	WCM053_	Y	Y	Y	Lung	Metastasis	Male	Never	Y	N	N	NA	58	95	Ba/Sq	Inflamed	37	0.533566
WCM053	WCM053_	Y	N	Y	Renal pelv	Primary	Male	Never	N	N	N	pT3	80	95	NA	NA	66	1.220329
WCM054	WCM054_	Y	Y	N	Renal pelv	Primary	Male	Former	Y	N	N	pT1	92	90	LumP	Depleted	17	0.182588
WCM054	WCM054_	Y	N	N	Renal pelv	Primary	Male	Former	N	N	N	pT1	85	80	NA	NA	17	0.121738
WCM056	WCM056_	Y	Y	N	Renal pelv	Primary	Female	Never	Y	N	N	pT0	97	90	LumP	Depleted	69	0.312863
WCM057	WCM057_	Y	N	N	Lymph noc	Metastasis	Female	Former	N	N	N	NA	67	80	NA	NA	87	1.949829
WCM065	WCM065_	Y	N	N	Ureter	Primary	Male	Former	N	N	Y	pT1	85	70	NA	NA	544	0.046913
WCM066	WCM066_	Y	N	N	Ureter	Primary	Male	Former	N	N	Y	pT3	27	60	NA	NA	328	0.035271
WCM068	WCM068_	Y	Y	N	Adrenal gl	Metastasis	Male	Former	N	N	N	NA	84	90	LumP	Depleted	366	0.217699
WCM068	WCM068_	Y	Y	Y	Peritoneur	Metastasis	Male	Former	N	N	N	NA	74	90	LumP	Depleted	211	2.641418
WCM068	WCM068_	Y	Y	Y	Renal pelv	Primary	Male	Former	N	N	N	pT4	87	80	LumP	Depleted	364	0.231907
WCM071	WCM071_	Y	N	N	Renal pelv	Primary	Male	Former	N	N	Y	pT3	84	80	NA	NA	713	0.175422
WCM071	WCM071_	Y	N	N	Ureter	Primary	Male	Former	N	N	Y	pT3	78	80	NA	NA	699	0.181536
WCM071	WCM071_	Y	N	N	Renal pelv	Primary	Male	Former	N	N	Y	pT3	80	80	NA	NA	643	0.192986
WCM072	WCM072_	Y	N	N	Ureter	Primary	Male	Former	N	N	N	pT3	61	70	NA	NA	109	0.84292
WCM073	WCM073_	Y	N	N	Renal pelv	Primary	Male	Former	Y	N	N	pTa	65	90	NA	NA	43	0.076014
WCM075	WCM075_	Y	Y	N	Renal pelv	Primary	Female	Never	N	N	N	pT1	98	90	LumP	Depleted	97	0.66138
WCM078	WCM078_	Y	Y	Y	Bone	Metastasis	Male	Former	N	N	N	NA	72	80	LumU	Inflamed	508	7.010704
WCM078	WCM078_	Y	N	N	Retroperit	Local recu	Male	Former	N	N	N	NA	55	60	NA	NA	444	4.921816
WCM079	WCM079_	Y	Y	Y	Lung	Metastasis	Female	Former	Y	N	Y	NA	19	60	LumP	Depleted	35	0.011681
WCM080	WCM080_	Y	N	N	Ureter	Primary	Male	Never	Y	N	N	pT1	47	40	NA	NA	60	0.266575
WCM081	WCM081_	Y	N	N	Renal pelv	Primary	Male	Former	N	N	Y	pT3	84	90	NA	NA	82	0.211311
WCM082	WCM082_	Y	N	N	Retroperit	Local recu	Male	Former	N	N	N	NA	26	40	NA	NA	47	0.05952
WCM083	WCM083_	Y	N	N	Lymph noc	Metastasis	Female	Never	N	N	N	NA	37	30	NA	NA	36	0.022151
WCM084	WCM084_	Y	N	N	Renal pelv	Primary	Female	Former	N	N	N	pT2	56	80	NA	NA	50	0.217185
WCM085	WCM085_	N	Y	Y	Lymph noc	Metastasis	Male	Former	N	N	N	NA	NA	80	Ba/Sq	Inflamed		
WCM085	WCM085_	N	Y	Y	Renal pelv	Primary	Male	Former	N	N	N	pT4	NA	80	Ba/Sq	Inflamed		

Supplementary Table 2. Immune-related genes used by RNA-seq based classifier

MIR650	*NCF2*	*MPEG1*
IGJ	*CYBB*	*HAVCR2*
IGLL5	*VSIG4*	*RASSF4*
CXCL10	*GBP5*	*NCKAP1L*
CXCL9	*SPI1*	*IL10RA*
LYZ	*CCL19*	*MSR1*
UBD	*FOLR2*	*F13A1*
C1S	*IL7R*	*SASH3*
C1QB	*CSF1R*	*SLAMF8*
CXCL13	*SLC15A3*	*SLA*
C1QC	*LTB*	*LST1*
HLA-DRB1	*HLA-DMA*	*GZMK*
HLA-DRA	*CD37*	*BCL2A1*
HLA-DRB5	*CCL3*	*CYTIP*
CCL21	*TREM2*	*LAG3*
C1QA	*MS4A6A*	*CIITA*
HLA-DQA1	*CD7*	*FERMT3*
FCGR3A	*FYB*	*DOK2*
C1R	*SLCO2B1*	*PILRA*
HLA-DPA1	*WARS*	*IL4I1*
CD74	*MS4A4A*	*GZMH*
HLA-DQB1	*AIF1*	*TRPV2*
SERPING1	*CST7*	*CCR1*
HLA-DQA2	*FPR1*	*SAMSN1*
TMEM176A	*LY96*	*IGSF6*
TMEM176B	*MAFB*	*SLC7A7*
CCL5	*C3AR1*	*CD86*
HLA-DMB	*PTPRC*	*WAS*
RGS1	*ACP5*	*STAB1*
FCGR2A	*CD4*	*CD300A*
CD14	*HCK*	*SPOCK2*
HLA-DRB6	*LCP2*	*CD8A*
FCER1G	*CLEC7A*	*S100B*
HLA-DPB1	*EVI2B*	*TNFSF13B*
IDO1	*SDS*	*ARHGAP9*
IFI30	*HLA-DQB2*	*EVI2A*
ITGB2	*FGL2*	*CYTH4*
GZMB	*SELPLG*	*ADAP2*
TYROBP	*C5AR1*	*ABI3*
IL2RG	*FAM26F*	*ITGAX*
LAPTM5	*HLA-DOA*	*RCSD1*
C2	*PLEK*	*TBXAS1*
CCL4	*GPR183*	*LY86*
SRGN	*ELK2AP*	*DOCK2*
CD52	*PLA2G7*	*MYO1F*
FPR3	*CD79A*	*PIK3AP1*
RNASE1	*WIPF1*	*PTPN7*
CD53	*RNASE6*	*ITGAM*
MZB1	*CD48*	*ADAMDEC1*
CD163	*HCST*	*OSCAR*
CXCL11	*CORO1A*	*HK3*
ALOX5AP	*SLAMF7*	*LAT2*
CD3E	*C1orf162*	
CPVL	*PRF1*	
GNLY	*MNDA*	
NKG7	*CCL13*	
CD2	*LAIR1*	
GZMA	*IL2RB*	
CD3D	*IRF8*	

Supplementary Table 3. A list of the metal-labeled antibodies used in Imaging mass cytometry.

Metal tag	Antibody	Clone	Dilution	Stock concentration (mg/ml)	Vendor	Catalog	Citations
113In	Total Histone H3	D1H2	1:400	0.5	Cell Signaling Technology	4499BF	#1
141Pr	Alpha-smooth muscle actin (SMA)	1A4	1:400	0.5	Fluidigm	3141017D	#2
143Nd	Vimentin	D21H3	1:100	0.5	Fluidigm	3143027D	#1
144Nd	CD206	E2L9N	1:100	0.2	Cell Signaling Technology	91992BF	#3
146Nd	CD16	EPR16784	1:100	0.5	Fluidigm	3146020D	#2
147Sm	CD163	EDHu-1	1:100	0.5	Fluidigm	3147021D	#2
148Nd	Pan-Keratin	C11	1:100	0.5	Fluidigm	3148020D	#2
149Sm	CD11b	SP331	1:50	0.5	Abcam	ab238794	#4
150Nd	PD-1	D4W2J	1:50	0.5	Cell Signaling Technology	86163BF	#5
151Eu	CD31	EP3095	1:50	0.5	Abcam	ab216459	#1
152Sm	CD45	D9M8I	1:50	0.5	Fluidigm	3152018D	#2
153Eu	GATA3	D13C9	1:25	0.5	Cell Signaling Technology	5852BF	#6
155Gd	FoxP3	236A/E7	1:25	0.5	Invitrogen	14-4777-82	#7
156Gd	CD4	OTI5D9	1:50	0.5	Novus Biologicals	NBP2-70357	#7
158Gd	E-Cadherin	24E10	1:50	0.5	Fluidigm	3158029D	#2
159Tb	CD68	KP1	1:50	0.5	Abcam	ab233172	#1
161Dy	CD20	L26	1:200	0.5	Novus Biologicals	NBP2-80486	#2
162Dy	CD8a	C8/144B	1:100	0.5	eBioscience	14-0085-82	#1
163Dy	KRT5	EP1601Y	1:200	0.5	Abcam	ab214586	#1
167Er	GranzymeB	EPR20129-217	1:50	0.5	Fluidigm	3167021D	#2
168Er	Ki-67	B56	1:50	0.5	BD Pharmingen	556003	#1
169Tm	collagen type I	Polyclonal	1:300	0.5	Fluidigm	3169023D	#2
170Er	CD3	Polyclonal, C-Terminal	1:100	0.5	Fluidigm	3170019D	#2
173Yb	CD45RO	UCHL1	1:100	0.5	Invitrogen	14-0457-82	#5
175Lu	PD-L1	SP142	1:25	0.5	Abcam	ab236238	#7
176Yb	CD11c	EP1347Y	1:50	0.5	Abcam	ab216655	#5

#1 Kuett, L. et al. Three-dimensional imaging mass cytometry for highly multiplexed molecular and cellular mapping of tissues and the tumor microenvironment. Nat Cancer 3, 122–133, doi:10.1038/s43018-021-00301-w (2022).

#2 Chang Q, Ornatsky O, Hedley D. Staining of frozen and formalin-fixed, paraffin-embedded tissues with metal-labeled antibodies for Imaging Mass Cytometry analysis. Current Protocols in Cytometry 82: 12.47.1–12.47.8, doi: 10.1002/cpcy.29 (2017).

#3 Rendeiro, A. F. et al. The spatial landscape of lung pathology during COVID-19 progression. Nature 593, 564-569, doi:10.1038/s41586-021-03475-6 (2021).

#4 Li B et al. Myeloid-Derived Suppressive Cells Deficient in Liver X Receptor α Protected From Autoimmune Hepatitis. Front Immunol 12:732102, doi: 10.3389/fimmu.2021.732102 (2021).

#5 Ijsselsteijn ME, van der Breggen R, Farina Sarasqueta A, Koning F, de Miranda NFCC. A 40-Marker Panel for High Dimensional Characterization of Cancer Immune Microenvironments by Imaging Mass Cytometry. Front Immunol. 10:2534, doi:10.3389/fimmu.2019.02534 (2019).

#6 https://antibodyregistry.org/search.php?q=AB_10835690

#7 Alnajar H. et al. Tumor-immune microenvironment revealed by Imaging Mass Cytometry in a metastatic sarcomatoid urothelial carcinoma with a prolonged response to pembrolizumab. Cold Spring Harb Mol Case Stud. 8:a006151, doi:10.1101/mcs.a006151 (2022).

Supplementary Table 4: Exome sequencing statistic summaries

Sample ID	total reads	percentage of captured reads	average coverage
WCM006_P	84,367,815	88.87%	111
WCM006_Ctrl	83,197,035	86.26%	107
WCM010_P	86590826	85.75%	112
WCM010_M1	82,936,503	84.22%	104
WCM010_M2	85014281	85.53%	110
WCM010_M3	82315255	85.69%	107
WCM010_Ctrl	78193110	84.41%	99
WCM016_P	72,996,642	94.01%	99
WCM016_M	88,162,658	85.80%	114
WCM016_Ctrl	84,480,477	85.34%	108
WCM018_M	77,972,152	91.87%	105
WCM018_Ctrl	87,348,284	87.72%	114
WCM019_P	88,179,162	86.92%	112
WCM019_Ctrl	103,376,657	83.02%	128
WCM022_P	68,180,823	87.17%	86
WCM022_Ctrl	72,038,258	84.10%	88
WCM031_P	71,372,403	88.14%	92
WCM031_M1	65,823,429	82.91%	81
WCM031_M2	49,836,783	81.30%	60
WCM031_Ctrl	69,805,994	83.13%	86
WCM045_P	59,798,228	96.24%	80
WCM045_M	45,846,798	91.96%	60
WCM045_Ctrl	64,647,599	85.53%	82
WCM052_P	55,916,692	88.34%	72
WCM052_M	51,947,475	88.96%	68
WCM052_Ctrl	70,295,221	86.44%	89
WCM053_P	75,181,046	86.04%	94
WCM053_M	68,513,098	86.04%	84
WCM053_Ctrl	76,647,892	86.22%	96
WCM054_P1	57,899,049	87.13%	72
WCM054_P2	61,323,016	91.09%	80
WCM054_Ctrl	64,838,218	87.06%	83
WCM056_P	62,531,259	85.76%	79
WCM056_Ctrl	59,928,390	87.98%	77
WCM057_M	59,963,511	84.60%	76
WCM057_Ctrl	62,252,544	85.68%	79
WCM065_P	77,336,831	93.99%	105
WCM065_Ctrl	68,147,938	88.32%	88
WCM066_P	73,332,007	94.25%	98
WCM066_Ctrl	68,228,930	88.94%	89
WCM068_P	66,064,418	86.16%	85
WCM068_M1	65,501,644	86.74%	85
WCM068_M2	88361510	83.45%	111
WCM068_Ctrl	61,576,487	86.94%	79
WCM071_P1	80,881,863	93.95%	110
WCM071_P2	79,837,841	94.25%	108
WCM071_P3	80,014,698	94.02%	108
WCM071_Ctrl	92,992,241	86.43%	120
WCM072_P	91114638	88.56%	119
WCM072_Ctrl	83937104	85.02%	108
WCM073_P	87,215,644	91.75%	117
WCM073_Ctrl	70,477,833	88.58%	93
WCM075_P	73,932,941	83.43%	92
WCM075_Ctrl	72,796,833	85.55%	93
WCM078_M1	86,440,090	87.17%	113
WCM078_M2	82,265,799	84.24%	104
WCM078_Ctrl	84600526	86.16%	109
WCM079_M	82,192,206	83.30%	103
WCM079_Ctrl	85,825,011	90.58%	116
WCM080_P	68,189,832	87.13%	87
WCM080_Ctrl	64,393,280	90.53%	84
WCM081_P	67,603,930	89.66%	89
WCM081_Ctrl	63,186,802	82.19%	77
WCM082_M	86,601,506	87.57%	113
WCM082_Ctrl	90,466,937	77.72%	106
WCM083_M	99,131,758	78.25%	117
WCM083_Ctrl	89,341,769	77.08%	105
WCM084_P	108,957,788	83.06%	136
WCM084_Ctrl	102,564,963	80.65%	125
