# Supplementary material for: The evolution of metastatic upper tract urothelial carcinoma through genomic-transcriptomic and single-cell protein markers analysis
Source: Nat Commun. 2024 Mar 18;15:2009. doi: 10.1038/s41467-024-46320-w (PMC10948878; doi:10.1038/s41467-024-46320-w)
Supplement: Supplementary file 3 — Description of Additional Supplementary Files [file 41467_2024_46320_MOESM3_ESM.pdf]

Description of Additional Supplementary Files

Supplementary Data 1 : A list of all non-synonymous mutations and copy number alterations detected by WES
