# Supplementary material for: The evolution of metastatic upper tract urothelial carcinoma through genomic-transcriptomic and single-cell protein markers analysis
Source: Nat Commun. 2024 Mar 18;15:2009. doi: 10.1038/s41467-024-46320-w (PMC10948878; doi:10.1038/s41467-024-46320-w)
Supplement: Supplementary file 5 — Reporting Summary [file 41467_2024_46320_MOESM5_ESM.pdf]

Corresponding author(s): Bishoy M. Faltas
Juan Miguel Mosquera

Last updated by author(s): Feb 12, 2024

Reporting Summary

Nature Portfolio wishes to improve the reproducibility of the work that we publish. This form provides structure for consistency and transparency in reporting. For further information on Nature Portfolio policies, see our Editorial Policies and the Editorial Policy Checklist.

Statistics

For all statistical analyses, confirm that the following items are present in the figure legend, table legend, main text, or Methods section.

n/a	Confirmed	
☐	☒	The exact sample size (*n*) for each experimental group/condition, given as a discrete number and unit of measurement
☐	☒	A statement on whether measurements were taken from distinct samples or whether the same sample was measured repeatedly
☐	☒	The statistical test(s) used AND whether they are one- or two-sided<br>*Only common tests should be described solely by name; describe more complex techniques in the Methods section.*
☐	☒	A description of all covariates tested
☐	☒	A description of any assumptions or corrections, such as tests of normality and adjustment for multiple comparisons
☐	☒	A full description of the statistical parameters including central tendency (e.g. means) or other basic estimates (e.g. regression coefficient) AND variation (e.g. standard deviation) or associated estimates of uncertainty (e.g. confidence intervals)
☐	☒	For null hypothesis testing, the test statistic (e.g. *F*, *t*, *r*) with confidence intervals, effect sizes, degrees of freedom and *P* value noted<br>*Give P values as exact values whenever suitable.*
☒	☐	For Bayesian analysis, information on the choice of priors and Markov chain Monte Carlo settings
☒	☐	For hierarchical and complex designs, identification of the appropriate level for tests and full reporting of outcomes
☐	☒	Estimates of effect sizes (e.g. Cohen's *d*, Pearson's *r*), indicating how they were calculated

Our web collection on statistics for biologists contains articles on many of the points above.

Software and code

Policy information about availability of computer code

Data collection	OncoKB database was used for determination of pathogenicity and actionability for each detected mutation and CNA.<br>The expression data for TCGA bladder tumors were obtained from the GDC/TCGA bladder cohort.<br>No software was used for data collection.
Data analysis	All the WCM samples data were processed through the computational analysis pipeline of the Institute for Precision Medicine at Weill Cornell, New York Presbyterian Hospital (IPM-Exome-pipeline [v0.9]). Raw reads quality was assessed with FASTQC.<br>For RNA sequencing analysis of WCM UTUC tumors, all reads were independently aligned with STAR_2.4.0f1 for sequence alignment against the human genome sequence build hg19, downloaded via the UCSC genome browser (http://hgdownload.soe.ucsc.edu/goldenPath/hg19/bigZips/), and SAMTOOLS v0.1.19 for sorting and indexing reads. Cufflinks (2.0.2) was used to estimate the expression values (FPKMS), and GENCODE v23 GTF file for annotation. Rstudio with R (v3.6.1) was used for the statistical analysis and the generation of figures.<br>MSI score was calculated by the MSI sensor (https://github.com/ding-lab/msisensor).<br>ConsensusMIBC R package was used to infer molecular subtypes of urothelial carcinoma.<br>The following software/tools/algorithms/ packages were used: ilastik (version 1.3.3) for analysis of Imaging Mass Cytometry™ (IMC) data: ilastik (version 1.3.3) , DeepCell (version 0.8.2), skimage.measure.regionprops_table function (version 0.18.1), Scanpy (version 1.7.1), batch balanced k-nearest neighbors (bbknn) (version 1.4.0), umap package (version 0.4.6), leidenalg package (version 0.8.3)

For manuscripts utilizing custom algorithms or software that are central to the research but not yet described in published literature, software must be made available to editors and reviewers. We strongly encourage code deposition in a community repository (e.g. GitHub). See the Nature Portfolio guidelines for submitting code & software for further information.

Data

Policy information about availability of data

All manuscripts must include a data availability statement. This statement should provide the following information, where applicable:

- Accession codes, unique identifiers, or web links for publicly available datasets
- A description of any restrictions on data availability
- For clinical datasets or third party data, please ensure that the statement adheres to our policy

The raw sequence data that support the findings of this study are available in the database of Genotypes and Phenotypes (dbGaP) under the accession number: phs001087.v3.p1 [https://www.ncbi.nlm.nih.gov/projects/gap/cgi-bin/study.cgi?study_id=phs001087.v3.p1].For IMC™, the pre-processed .tiff files are available at https://doi.org/10.5281/zenodo.5719188. Source data are provided with this paper.

Research involving human participants, their data, or biological material

Policy information about studies with human participants or human data. See also policy information about sex, gender (identity/presentation), and sexual orientation and race, ethnicity and racism.

Reporting on sex and gender	Data on sex was collected as part of this study and mentioned in Supplementary Table 1. However, this information was not used as a criterion for study design or data interpretation. Information on gender was not collected.
Reporting on race, ethnicity, or other socially relevant groupings	Socially relevant/constructed categorization was not used in this study.
Population characteristics	Patients with a pathologically confirmed diagnosis of upper tract urothelial carcinoma (UTUC) were selected. A total of 44 UTUC from 28 patients with high-grade UTUC were included in this cohort. Of these, 25 samples were primary and 19 samples were metastatic/local recurrence. Twenty seven samples were collected from chemotherapy-naïve patients, and 17 samples from chemotherapy-treated patients.
Recruitment	Patients were recruited to tissue banks for Precision Medicine Study under institutional ethical-approved protocols with written informed consent.
Ethics oversight	The study protocol was approved by the Weill Cornell Institutional Review Board (IRB No. 1305013903).

Note that full information on the approval of the study protocol must also be provided in the manuscript.

Field-specific reporting

Please select the one below that is the best fit for your research. If you are not sure, read the appropriate sections before making your selection.

☒ Life sciences ☐ Behavioural & social sciences ☐ Ecological, evolutionary & environmental sciences

For a reference copy of the document with all sections, see nature.com/documents/nr-reporting-summary-flat.pdf

Life sciences study design

All studies must disclose on these points even when the disclosure is negative.

Sample size	No statistical method for sample size determination was performed. The samples were included based on sample availability. Imaging mass cytometry was performed on all tumors whose archived FFPE tissue was available.
Data exclusions	No data were excluded from the analyses.
Replication	No technical replicates were performed since WES, RNA-seq and IMC data were obtained from individual samples.
Randomization	Randomization is not relevant as no allocation to experimental groups were performed.
Blinding	Blinding is not relevant as the knowledge of the samples characteristics was needed to assign them to specific groups.

Reporting for specific materials, systems and methods

We require information from authors about some types of materials, experimental systems and methods used in many studies. Here, indicate whether each material, system or method listed is relevant to your study. If you are not sure if a list item applies to your research, read the appropriate section before selecting a response.

Materials & experimental systems

n/a	Involved in the study
☐	☒ Antibodies
☒	☐ Eukaryotic cell lines
☒	☐ Palaeontology and archaeology
☒	☐ Animals and other organisms
☒	☐ Clinical data
☒	☐ Dual use research of concern
☒	☐ Plants

Methods

n/a	Involved in the study
☒	☐ ChIP-seq
☒	☐ Flow cytometry
☒	☐ MRI-based neuroimaging

Antibodies

Antibodies used

Metal tag Antibody Clone Dilution Stock concentration (mg/ml) Vendor Catalog
113In Total Histone H3 D1H2 1:400 0.5 Cell Signaling Technology 4499BF
141Pr Alpha-smooth muscle actin (SMA) 1A4 l:400 0.5 Fluidigm 3141017D
143Nd Vimentin D21H3 1:100 0.5 Fluidigm 3143027D
144Nd CD206 E2L9N 1:100 0.2 Cell Signaling Technology 91992BF
146Nd CD16 EPR16784 1:100 0.5 Fluidigm 3146020D
147Sm CD163 EDHu-1 1:100 0.5 Fluidigm 3147021D
148Nd Pan-Keratin Cll 1:100 0.5 Fluidigm 3148020D
149Sm CDllb SP331 l:50 0.5 Abeam ab238794
150Nd PD-1 D4W2J 1:50 0.5 Cell Signaling Technology 86163BF
151Eu CD31 EP3095 1:50 0.5 Abeam ab216459
152Sm CD45 D9M8I l:50 0.5 Fluidigm 3152018D
153Eu GATA3 D13C9 1:25 0.5 Cell Signaling Technology 5852BF
155Gd FoxP3 236A/E7 1:25 0.5 lnvitrogen 14-4777-82
156Gd CD4 OTl5D9 l:50 0.5 Novus Biologicals NBP2-70357
158Gd E-Cadherin 24El0 1:50 0.5 Fluidigm 3158029D
159Tb CD68 KPl 1:50 0.5 Abeam ab233172
161Dy CD20 L26 1:200 0.5 Novus Biologicals NBP2-80486
162Dy CD8a C8/144B 1:100 0.5 eBioscience 14-0085-82
163Dy KRT5 EP1601Y 1:200 0.5 Abeam ab214586
167Er GranzymeB EPR20129-217 1:50 0.5 Fluidigm 3167021D
168Er Ki-67 B56 1:50 0.5 BD Pharmingen 556003
169Tm collagen type I Polyclonal 1:300 0.5 Fluidigm 3169023D
l70Er CD3 Polyclonal, C-Terminal 1:100 0.5 Fluidigm 3170019D
173Yb CD45RO UCHLl 1:100 0.5 lnvitrogen 14-0457-82
l75Lu PD-Ll SP142 1:25 0.5 Abeam ab236238
l76Yb CDllc EP1347Y 1:50 0.5 Abeam ab216655

Validation

Antibodies were validated on appropriate controls using IHC, as presented on the manufacturer's datasheet. Custom conjugated clones were internally validated using IHC and verified by a pathologist. Moreover, the results of IMC performed at our institution have been published at multiple peer-reviewed journals (Cold Spring Harb Mal Case Stud. 2022 Apr 28;8(3):a006151., Nature. 2021 May;593(7860):564-569). Validation of the antibodies has been described in the main text of the manuscript and relevant literature citations are provided in Supplemental Table 3.
